# Pre- and post-pyrolysis effects on iron impregnation of ultrasound pre-treated softwood biochar for potential catalysis applications

**DOI:** 10.1007/s42452-021-04636-y

**Published:** 2021-05-17

**Authors:** Aneeshma Peter, Bruno Chabot, Eric Loranger

**Affiliations:** I2E3 – Institut d’Innovations en Écomatériaux, Écoproduits et Écoénergies, à base de biomasse, Université du Québec à Trois-Rivières, 3351, Boul. des Forges, Trois-Rivières, QC G8Z 4M3 Canada

**Keywords:** Softwood biochar, Iron impregnation, Slow pyrolysis, Ultrasound pre-treatments, Physicochemical properties

## Abstract

Slow pyrolysis is widely used to convert biomass into useable form of energy. Ultrasound pre-treatment assisted pyrolysis is a recently emerging methodology to improve the physicochemical properties of products derived. Biochar, the solid residues obtained from pyrolysis, is getting considerable attention because of its good physicochemical properties. Various modification techniques have been implemented on biochars to enhance their properties. Ultrasonic pre-treated wood biochar has showcased efficient surface and adsorption properties. Iron impregnated biochar is interesting as it has potentially proved the efficiency as an efficient low-cost catalyst. In this study, by combining the advantages of ultrasonic pre-treatment and iron impregnation, we have synthesized a series of Fe-impregnated biochar from softwood chips. Pre- and post-pyrolysis methods using a lab-scale pyrolyser had been implemented to compare the pyrolysis product yields and degree of impregnation. Biochars derived from ultrasound pre-treated woodchips by post pyrolysis demonstrated better impregnation of Fe ions on surface with better distribution of pyrolysis products such as biochar and biogas. The surface functionality of all ultrasound pre-treated biochars remained the same. However, post-pyrolysed samples at high frequency ultrasound pre-treatment showed better thermal stability. The chemical characteristics of these modified biochars are interesting and can indeed be used as a cost-effective replacement for various catalytic applications.

## Introduction

Efficient use of biomass into useable form of energy has always been a key concern by virtue of increasing energy demand, depletion of fossil fuel and global climate change [1–4]. Intense researches have been carried out globally to seek solutions for climate change by making use of renewable energy resources [5]. The province of Quebec in Canada, being rich in forest biomass availability, has engaged in adopting research policies to make use of the biomass products to address these challenges. Adequate techniques to process feedstock for better material quality and enhancing production methodologies to have potential materials for other applications are part of these venture. Slow pyrolysis, being a technique to convert biomass to different energy forms, has gained considerable attention because of its relative low-cost, efficiency and ease in processability [6]. The thermal decomposition of feedstocks under limited oxygen conditions result in forming liquid bio-oil, solid biochar, and non-condensable gas and are efficiently used for various applications. The properties of these products depend on several important parameters such as pyrolysis conditions, feedstock types, pre- and post-processing techniques on feedstocks [7–10].

Influence of power ultrasound treatments on pyrolysis and derived products have recently been explored. Power ultrasound or high intensity ultrasound make use of frequency range between 20 kHz and 1 MHz. The application of power ultrasound on biomass is based on the concept of cavitation. Formation of microjets during the implosion of cavitation bubbles disrupts the solid surface which is in contact and provides a unique physiochemical environment to the materials and thus, influences the material properties [11]. Understanding the ability of ultrasound to promote chemical and thermal decomposition reactions during pyrolysis with better efficiency in terms of product yield and physicochemical properties will be a key step to introduce ultrasound of biomass processing at industrial level. In one of the interesting studies carried out by Cherpozat et al. [12], it was shown that ultrasound pre-treatments on softwood chips resulted in enhanced bio-oil yield. A series of two ultrasound frequencies (170 kHz for 0.5 h followed by 40 kHz for 1.5 h) raised the bio-oil yield by 12 percent compared to untreated wood samples. Recently, Hazrati et al. [13] has shown the ability of ultrasonic pre-treatments to enhance the physicochemical characteristics and environmental applicability of sludge-derived biochar. Few other studies have also revealed the influence of ultrasonic treatments on improving adsorption properties of biochar produced from pyrolysis [14–17].

The intrinsic nature of the biomass feedstock and the processing techniques used play an important role in determining the properties of the biochar. Consequently, modification of biochar surfaces is an interesting approach to improve their physicochemical properties. Though activation is considered to be one among the efficient modification methods on biochar, introducing different composites on to the carbon surface is getting considerable attention because of the easy processability and efficiency. The resulting composites introduce completely new active sites on the carbon surface which has enhanced physicochemical properties suitable for various applications [18]. It has been shown that biochar modified with Fe oxides provides a selective affinity towards heavy metals like Cr, Eu and As [19–21]. They are also efficient for removing anionic contaminants from aqueous solutions [22]. Biochar supported metal oxides have also been studied for catalytic activity [23–26]. They have been used as efficient catalysts for biomass hydrolysis, dehydration and biodiesel production due to its tailoring properties and large surface area. A study by Ren and co-workers [27] revealed that the corn stover biochar catalyst enhanced the syngas and improved the bio-oil quality in biomass pyrolysis. Few other reports investigated the effect of metal impregnated biochar as catalyst for biomass gasification and the effect of such materials in upgrading process of bio-oils [28–30]. Kastner et al. [31] reported that the iron supported biochar could decompose toluene, a model tar compound, with linear increase in temperature and catalyst loading. They claimed that the inexpensive iron impregnated biochar catalysts could potentially be used to decompose tar molecules in syngas generated via biomass gasification. The catalytic activity of Fe-impregnated sugarcane biochar (FSB) for removing azo dye Orange G was investigated by Park et al. [32]. They have shown that the FSB was more economical, efficient, and recyclable than other conventional Fenton oxidation catalysts. Recently, Cao et al. [33], had proved the electrocatalytic activity of biochar made from iron enriched plants. Nejati et al. [34] has also investigated the upgradation of pyrolysis products using Fe based biochar as catalyst. They have stated that the energy recovery and process efficiency was achieved using biochar catalyst.

Metal impregnation experiments are generally carried out directly on the feedstock as a pre-treatment or on the biochar produced after the pyrolysis (post-treatment). The effect of these two processes on biochar can be varied. However, to our knowledge, no reports are available which examine the difference in biochar surface morphology caused by metal impregnation. In previous studies, we have shown that the surface morphology of biochar synthesized has already been altered by ultrasonication [14, 15]. The modification with metal oxides on these ultrasound pre-treated wood biochars could be more effective because of their exposed surface pores and microchannels. In this work, pre- and post- pyrolysis treatments on metal impregnated samples were carried out to understand the difference in characteristics of biochar-metal composites and pyrolysis product yield. The effect of ultrasound pre-treatment assisted metal impregnation on biochars were also explored. Finally, we hope that these engineered biochars will offer better properties and yield, opening up potential use for various catalytic applications.

## Materials and methods

### Biomass feedstock and ultrasound pre-treatments

The feedstock used to produce biochar is a mix of softwood mainly including pine, fir, spruce and larch. They are processed woodchips supplied by a local Canadian pulp and paper mill. The woodchips were ground to 5 by 5 mm sized needles and subjected to different ultrasonic pre-treatments. In our previous studies on ultrasonic pre-treated (UST) wood biochar, we have shown that the combination of frequency, power, bath temperature and exposure time significantly affects the surface morphology and properties of resulting biochar [15]. Hence, in this study we have used the maximum and minimum conditions of these parameters. Table 1 presents ultrasonic pre-treatment conditions performed. A 34 L Ultrasonic bath (model BT90 from Ultrasonic Power Corporation USA) is used to perform the pre-treatments in water. For each treatment, 200 g of wood chips were dipped into 4 L of deionized water in a weighed mesh bag, to ensure that the wood chips are completely submerged in water and homogenously treated by ultrasound. The pre-treated woodchips were dried in oven at 105 °C for 48 h.

**Table 1:** Ultrasonic pre-treatment conditions applied to wood chips

Sample	Frequency (kHz)	Power (W)	Temperature (°C)	Time (h)
Fe-UST1	40	1000	80	2
Fe-UST2	40	250	20	1
Fe-UST3	170	1000	80	2
Fe-UST4	170	250	20	1

### Lab-scale slow pyrolysis set up

**Fig. 1:**
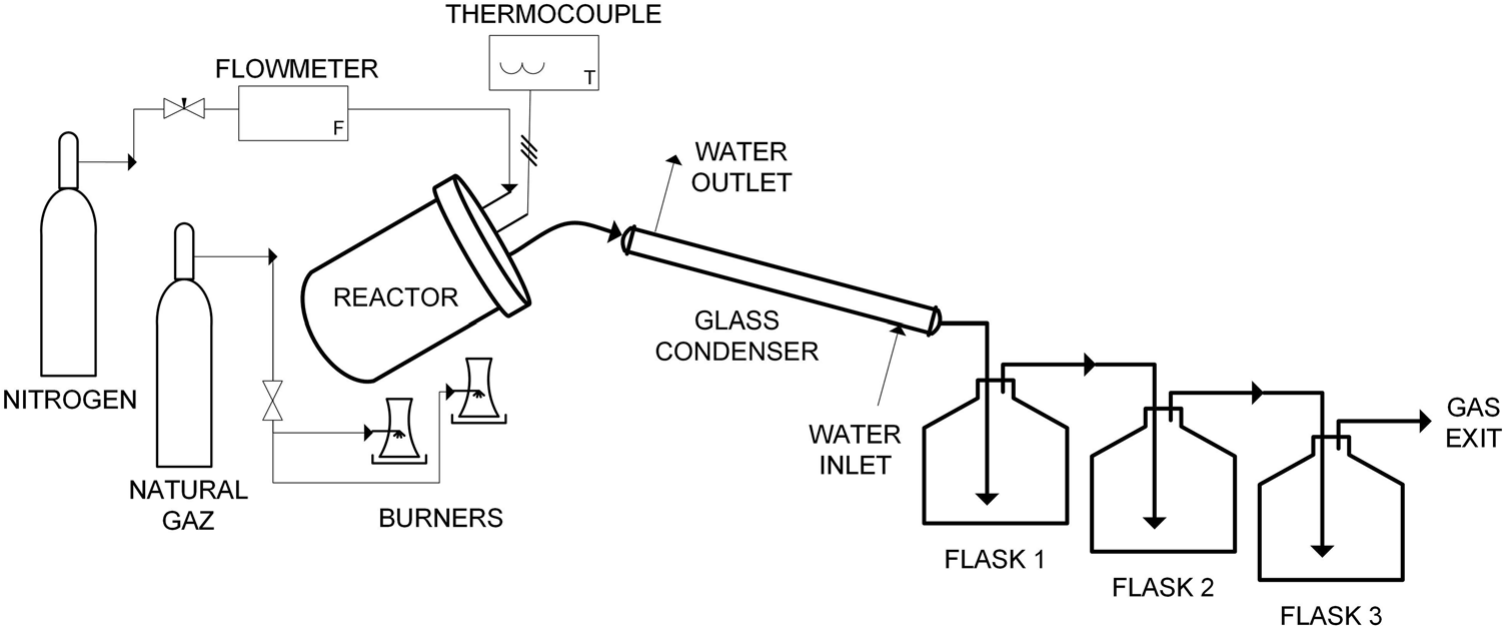
Schematic representation of laboratory-scale pyrolysis set up to produce biochar

The pyrolysis was performed using the method previously reported by Loranger et al. [35]. A schematic diagram of the pyrolysis system is given in Fig. 1. This laboratory scale system was originally developed for the production of bio-oil. The detailed experimental set-up is also described in the study of Cherpozat et al. [12], who mainly investigated the effect of different ultrasonic pre-treatments on bio-oil yield. However, the effect on biochar yield and properties were not analysed. The heating rate was approximately 15 °C/min and the peak temperature for the pyrolysis was 560 °C. The reaction was held at this temperature for about 1 h. From the heating rate and residence time (1–1.5 h), the system belongs to slow-pyrolysis category. At least three trials were performed on each treatment to confirm the average yield of biochar and other pyrolysis products. The pyrolysis products were collected separately, and the biochar was cleaned from tar using acetone and then stored in plastic bags.

### Metal impregnation

**Fig. 2:**
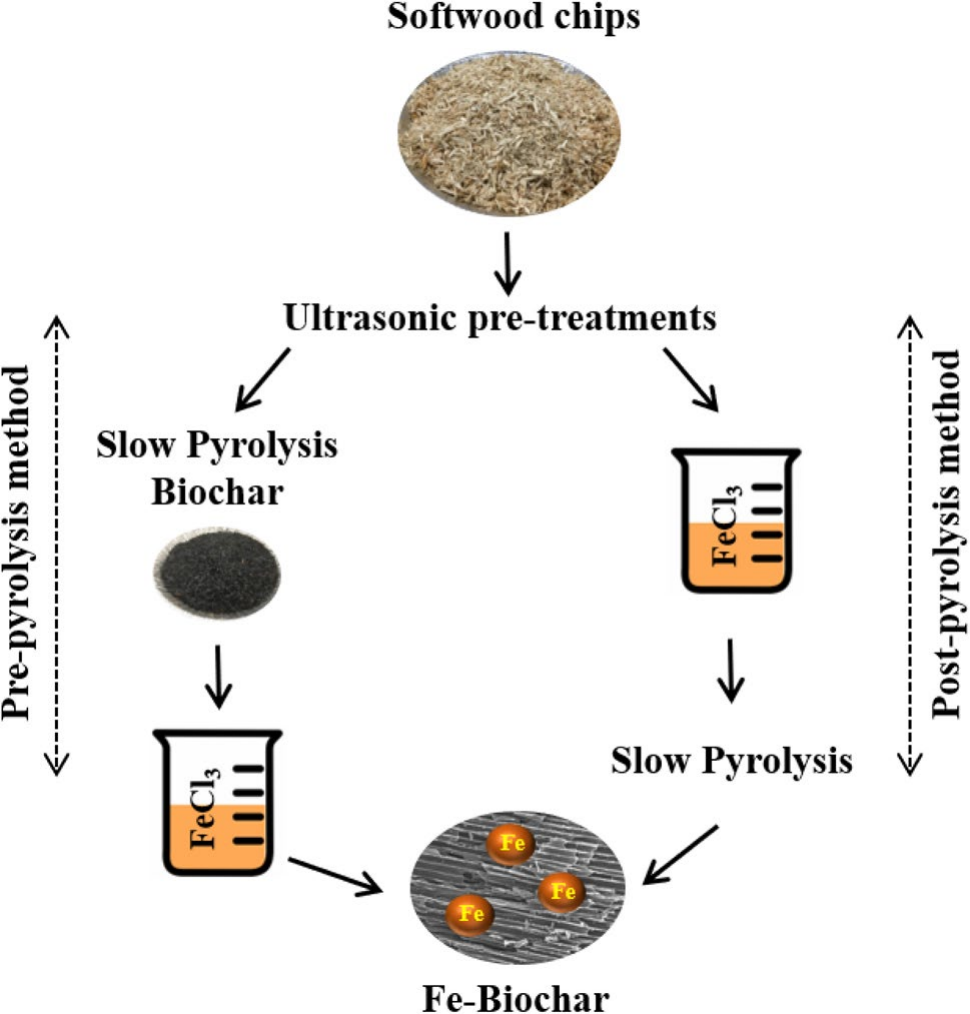
Schematic representation of Fe-impregnation methods

For metal impregnation, the method developed by Frišták et al. [19] was used. A 1:10 ratio (m:V) of sample was mixed with a 200 mmols ferric chloride solution. The mixture was stirred for 12 h under moderate heating (50 °C) and then dried at 105 °C for 24 h. The dried composites were rinsed several times with deionized water in order to remove free ions on the surface and then dried overnight at 105 °C. Pre and post-pyrolysis methods were designated based on iron impregnation step. In the pre-pyrolysis technique, iron was impregnated on to the biochar sample obtained after pyrolysis. However, in post-pyrolysis, iron impregnation was carried out on the ultrasound pre-treated woodchips followed by pyrolysis (Fig. 2).

### Surface and chemical characterisations

The surface functional groups were identified by infra-red spectroscopy (FTIR) using a Nicolet iS10 Smart iTR and KBr pellet. The reflection mode spectra were obtained in the range of 400–4000 cm^−1^ for a minimum of 32 scans with 4 cm^−1^ resolution. Scanning Electron Microscopy (SEM) (Hitachi SU1510) images of the materials were captured for surface morphology analysis. Energy Dispersive X-ray Spectroscopy (EDX) was obtained with an X-Max, Oxford instrument to verify the surface carbon and oxygen content. Thermogravimetric Analysis (TGA) was performed on a Perkin Elmer TGA 8000 Pyris series instrument. Under a flow of nitrogen (200 ml/min), the sample was heated from room temperature to 105 °C by ramping at 10 °C/min, and then heated to 800 °C.

## Results and discussion

### Effect of iron impregnation on pyrolysis product yields

**Fig. 3:**
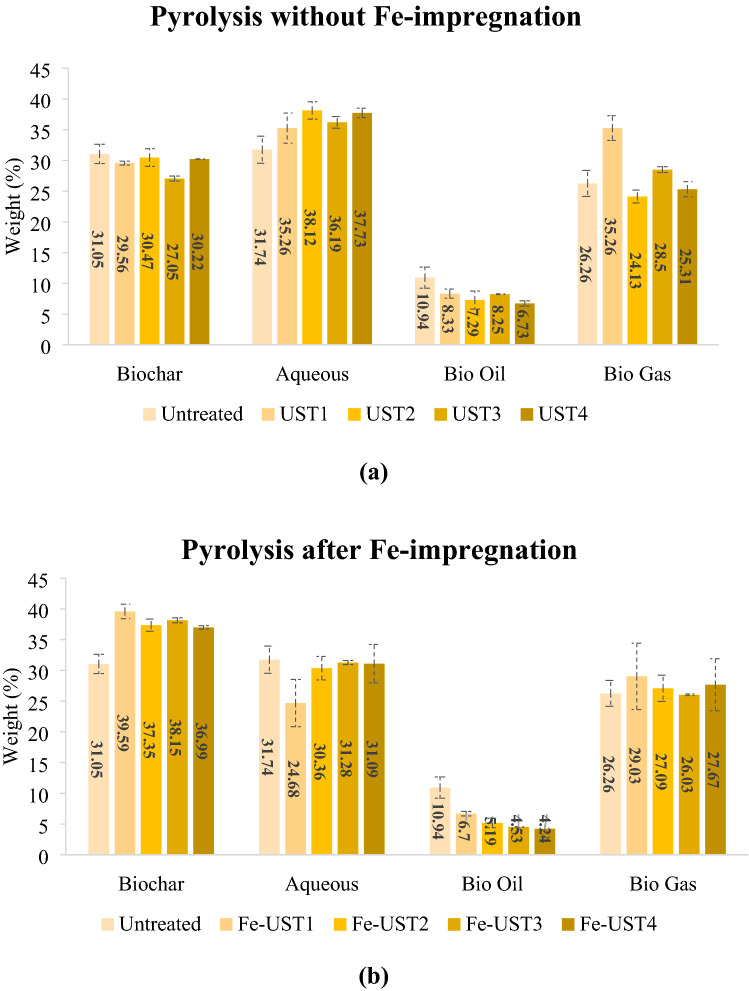
Pyrolysis product distribution of pre and post-pyrolysis methods **a** product yield for pyrolysis without Fe-Impregnation **b** product yield for pyrolysis after Fe-impregnation

To study the influence of ultrasonic pre-treatment assisted iron impregnation on biochars, pyrolysis product yields were estimated (Fig. 3). Fe-impregnation was done directly on the UST-woodchips, then subjected to pyrolysis (Post-pyrolysis) and the product yields were calculated and compared with corresponding non-impregnated sample yield. Figure 3a shows the product distribution of pyrolysis carried out on ultrasound pre-treated wood chips before Fe-impregnation. The percentage yield of biochar, aqueous phase obtained from condensation of gas, bio oil and biogas were compared to pyrolysis of a control feedstock (Untreated). The biochar and the liquid product yield (aqueous and bio-oil) was calculated directly from the initial and final weight difference of the containers. Biogas yield was calculated by mass balance (100-(biochar + aqueous + bio oil)). The UST-biochars exhibited the common trend which is dependent on pyrolysis temperature and reaction time. There was no significant change in yields with respect to ultrasonic pre-treatment conditions. However, a remarkable change in pyrolysis product yields were observed for post-pyrolysis method. As shown in Fig. 3b, the percentage yield of biochars increased significantly compared to the untreated sample. Concerning the related non-impregnated UST-biochars, this rise was more evident. Fe-UST1 has the maximum biochar yield among all with almost 33 percent increase compared to the corresponding non-impregnated sample (UST1). Despite of the increase in biochar yield, the bio-oil yield was dramatically decreased after Fe-impregnation. The pre-treated samples at 170 kHz (Fe-UST3 and Fe-UST4) had clearly lower yields compared to the untreated or non-impregnated UST-samples.

Dai et al. [29] has reported the same trend with bio oil. As explained in their study as well, the iron impregnated biochar formed during the pyrolysis itself act as a catalyst and facilitates the reforming or deoxygenation of pyrolysis vapors, forming the non-condensable gas. The primary reason for the increase in biogas percent during the post-pyrolysis method can be explained by this self-catalytic effect of iron impregnated biochars. Similarly, Yaman et al. [28] has also described the presence of metal on biochar due to the impregnation methods that shifted the deoxygenation mechanism of the catalyst from dehydration to decarbonylation and decarboxylation. They have reported a higher catalytic activity exhibited by Fe-impregnated SBA-15 with water, gas and solid product yields significantly enhanced.

These observations encouraged us to elucidate the efficiency of Fe-impregnated biochars for catalysis applications and how ultrasound pre-treatments could provide assistance to improve the impregnation on biochar surface.

### Effect of impregnation on biochar surface elemental composition

The surface elemental composition of modified biochars were analysed by EDX and the results are presented in Table 2.

**Table 2:** Atomic percent of different elements present on biochar surface

Sample	Pre-pyrolysis			Post-pyrolysis		
	C At%	O At%	Fe At%	C At%	O At%	Fe At%
Fe-UST1	87.9	10.5	1.1	95.4	3.3	1.3
Fe-UST2	89.1	10.0	0.5	91.7	7.1	0.7
Fe-UST3	90.6	7.0	1.4	88.8	8.7	1.5
Fe-UST4	89.1	9.5	1.0	90.8	7.3	1.5

EDX gives total atomic percentage of elements found on the surface and the results excludes the contribution of hydrogen. Samples at 170 kHz (UST 3 and 4) showed comparatively higher atomic percentages than samples at 40 kHz (Fe-UST 1 and 2). However, the increase was marginal. Fe-UST2 displayed the lowest impregnation rate among all in both pre- and post-pyrolysis methods. With respect to the higher oxygen content in pre-pyrolysis samples, the impregnation of Fe-ions was expected to be higher in these samples. Interestingly, post-pyrolysed samples exhibited slightly better atomic percentage of iron on the surface. The quantity of iron impregnation on UST modified biochars was better than those previously reported in the study by Dai et al. [29] in which they could achieve 0.15 percent of Fe^3+^ ions by using 0.2 mols of iron nitrate solution.

### Effect of impregnation on biochar surface morphology

**Fig. 4:**
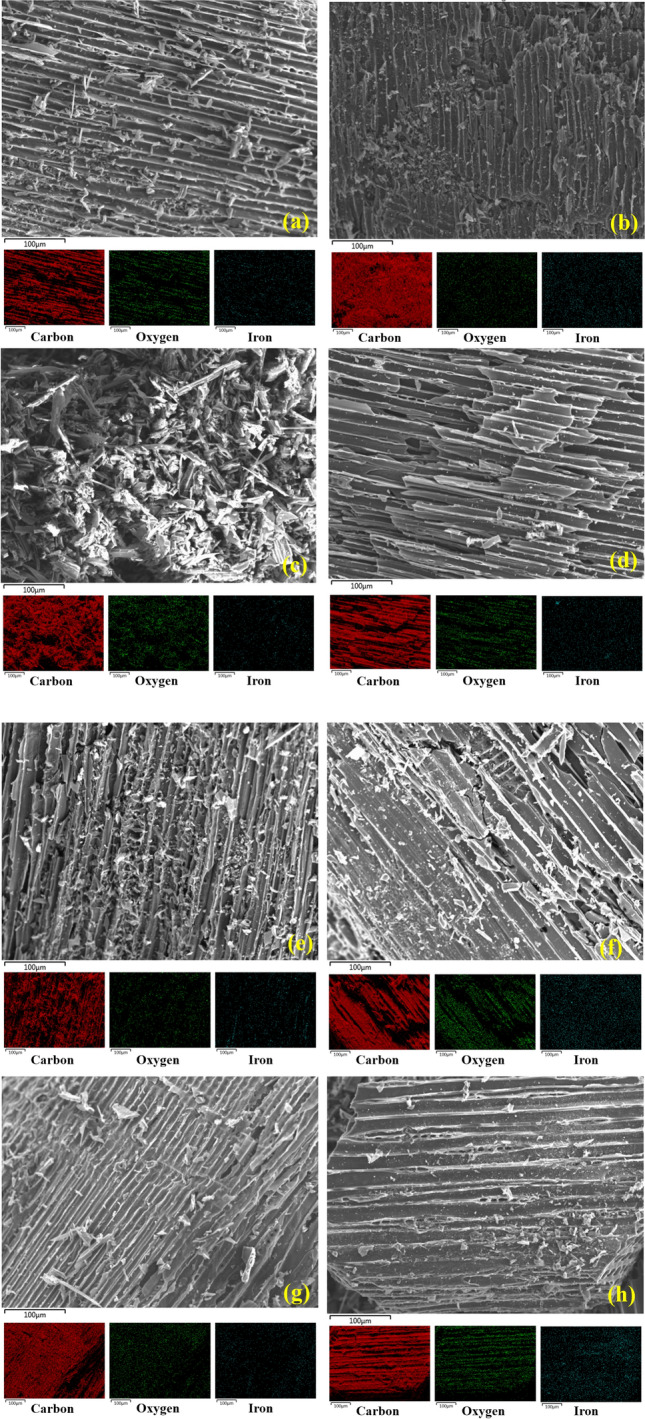
Scanning electron microscope and EDX mapping images of different ultrasonic pre-treated samples **a** and **b** pre and post pyrolysis biochar of Fe-UST1 **c** and **d** pre and post pyrolysis biochar of Fe-UST2 **e** and **f** pre and post pyrolysis biochar of Fe-UST3 **g** and **h** pre and post pyrolysis biochar of Fe-UST4

The morphology of the Fe-impregnated biochar samples prepared by pre- and post-pyrolysis was analyzed by SEM with magnification 250× (Fig. 4). The images mainly showed the difference in structural characteristics at each ultrasonic pre-treatment level.

In the case of pre-pyrolysis samples, the surface was more ruptured and heterogeneous with non-uniform channels and pores. This was more evident in the case of 40 kHz samples (Fe-UST1, Fe-UST2) and 170 kHz at high power (Fe-UST3). However, these alterations were less obvious in Fe-UST4 indicating that, at higher frequency and lower power, ultrasound pre-treatment did not really disturb the surface structure. For post-pyrolysed biochars, the microchannels and the layering were significantly altered, yet were consistent with the ultrasonic pre-treatments. Particularly, for low frequency samples (Fe-UST1 and Fe-UST2), the biochar surface seems to be more homogeneous and the microchannels were more polished compared to corresponding pre-pyrolysed samples. High frequency pre-treated samples (Fe-UST3 and Fe-UST4) were less affected and the surface was smooth. EDX mapping on localised surface indicated the homogenous spread of iron particles onto the surface. Post-pyrolysis samples demonstrated better distribution of Fe ions in comparison with pre-pyrolysis biochars. Among them, 170 kHz samples showed prominent distributions of Fe ions on the surface. The SEM and EDX results demonstrated the efficiency of ultrasonic pre-treatments to homogenously impregnate iron particles on the material surface.

### Effect of impregnation on biochar surface functional groups

**Fig. 5:**
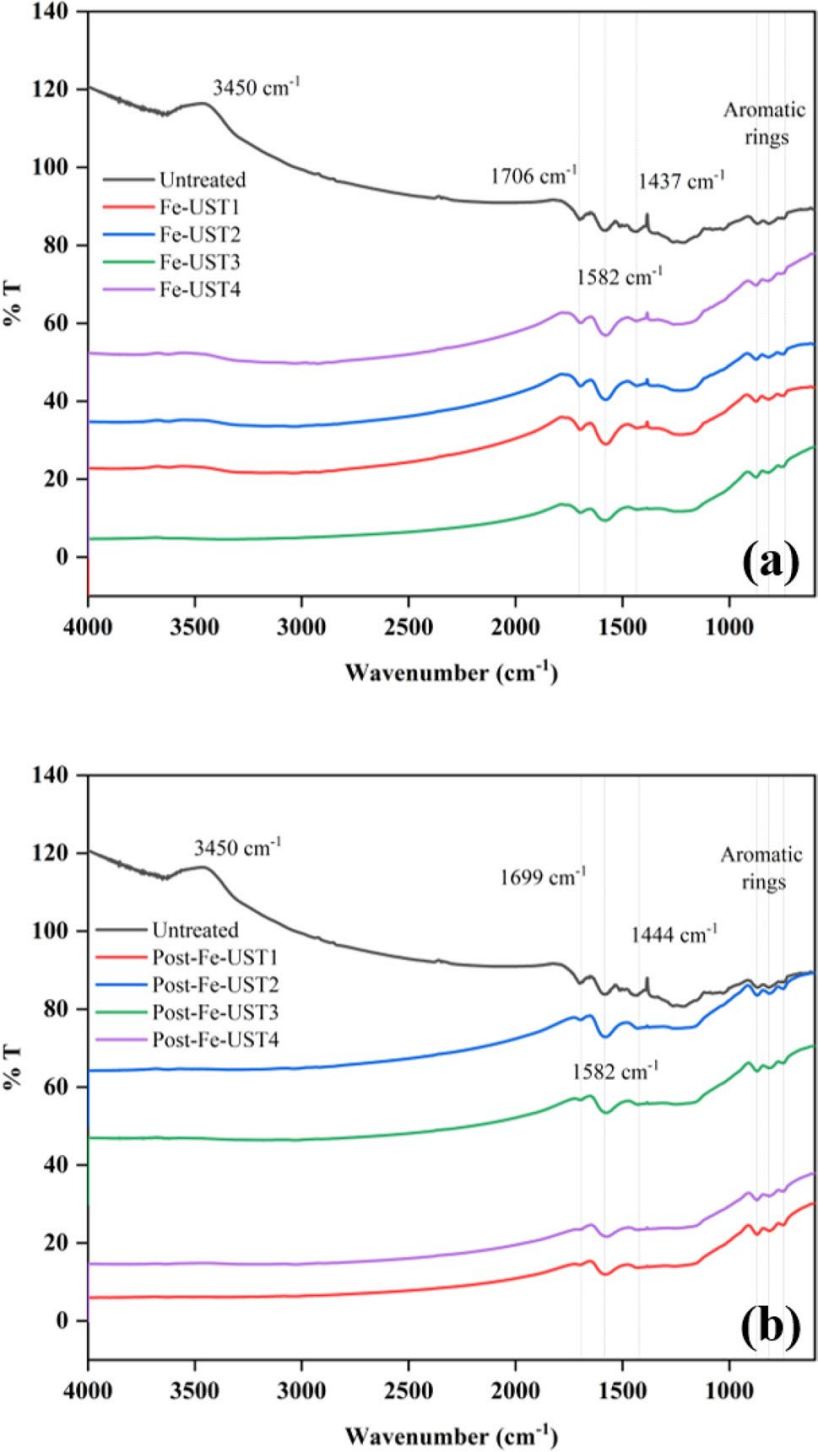
Infrared spectra of Fe-impregnated biochars prepared from **a** pre-pyrolysis methods **b** post-pyrolysis method

To understand the influence of Fe-impregnation aided by ultrasonic pre-treatment conditions on surface functionality of the biochar, infra-red spectra were analysed (Fig. 5). The O–H vibration peak observed at 3450 cm^−1^ disappeared after the impregnation experiments because of metal coordination with the functional group to form iron oxides. The main differences in FTIR spectra of unimpregnated and impregnated biochars were localized at wavelengths ranging from 1750 to 1250 cm^−1^. However, except for few intensity changes in bands, all the spectra were identical for every ultrasonic pre-treatment. At 1700 cm^−1^, C=O stretching vibrations of ketones and carboxylic acids were observed. Aromatic C–O stretching bands appeared at 1440 cm^−1^. A strong band at 1582 cm^−1^ indicates the C=C and C=O stretching vibrations of aryl groups and this peak appeared to be more intense after Fe-impregnation.

These results provide a better understanding of the fact that the chemical composition of biochar is not affected by ultrasound pre-treatments, even after Fe-impregnation. The characteristics peaks were comparable with previously reported results for iron impregnated biochars [19, 20, 22].

### Effect of iron impregnation on biochar thermal properties

**Fig. 6:**
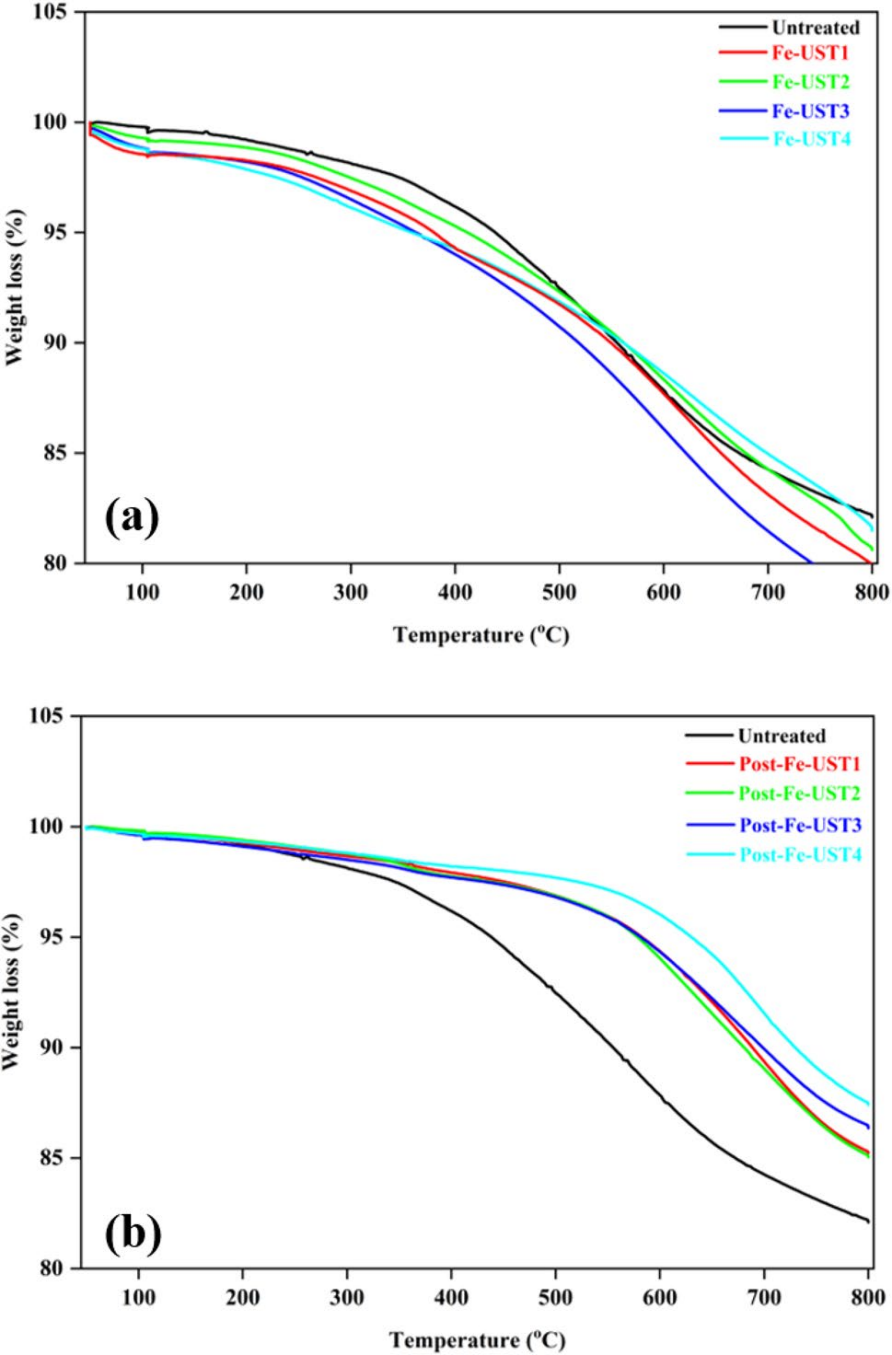
Thermogravimetric analysis of Fe-impregnated biochars prepared using (a) Pre-pyrolysis method (b) Post-pyrolysis method

Thermal stability of all synthesized biochars were analysed using Thermogravimetric analysis and Fig. 6 represents comparative study between pre- and post-pyrolysis biochars. As evident from the graph, post-pyrolysed biochar samples exhibited significantly better thermal stability than untreated or corresponding pre-pyrolysed samples. The samples were stable up to around 600 °C. High frequency pre-treated samples had slightly improved thermal stability and low power sample (Post-Fe-UST4) possessed the highest stable temperature. This can be due to the strong chemical bond formation between biochar surface and impregnated Fe particles [20], which further enhanced by high frequency ultrasonication in low power condition. For the pre-pyrolysed biochars, major weight loss happened at around 350 °C, which is much lower than the post-pyrolysis samples. Ultrasonic pre-treatments had nominal influence on the thermal stability of Fe-impregnated biochars and the stability was less in comparison with the untreated biochar. High power pre-treated samples, irrespective of frequency, were the least stable biochars among all.

The chemical characteristic of biochar synthesized by pre- and post-pyrolysis method indicated that, even though the surface functionality remains the same with ultrasonic pre-treatments and iron impregnation, the thermal stability of the material was greatly influenced by the impregnation method and ultrasonic conditions. These results demonstrate that, Fe-impregnation directly on biomass feedstock followed by pyrolysis provides biochar with higher yield, particle distribution on surface, and thermally much stable biochars. This low-cost, feasible methodology can be easily adapted in order to produce iron-based biochar catalysts to replace expensive and toxic metals like platinum, nickel etc.

## Conclusions

In this study, we have synthesized biochars modified with Fe ion particles using ultrasound assisted lab-scale slow pyrolysis. The lab-scale pyrolysis system could produce engineered biochars better yield exhibited after Fe-impregnation. post-pyrolysis method demonstrated better surface morphology with higher frequency samples and exhibited better impregnation results compared to low frequency samples. The surface functionality of all ultrasound pre-treated biochars remained the same in post and pre-pyrolysis method of impregnation. However, post-pyrolysed samples at high frequency ultrasound pre-treatment showed better thermal stability. These engineered biochars can be a potential, low-cost catalyst for various applications such as upgrading bio-oil, biodiesel production, hydrogen production, etc. Detailed investigations on bio-oil compositions produced with this technique and proof of catalytic activity of Fe-UST-biochars has yet to be done in order to examine potential applications.
